# Measuring the effect of environmental stress on inbreeding depression alone obscures the relative importance of inbreeding–stress interactions on overall fitness in *Callosobruchus maculatus*

**DOI:** 10.1111/eva.13060

**Published:** 2020-09-04

**Authors:** Amy L. Springer, Frank J. Messina, Zachariah Gompert

**Affiliations:** ^1^ Department of Biology Utah State University Logan UT USA; ^2^ Ecology Center Utah State University Logan UT USA

**Keywords:** *Callosobruchus maculatus*, environmental stress, hierarchical Bayesian models, inbreeding depression, population management

## Abstract

Environmental stress can have a profound effect on inbreeding depression. Quantifying this effect is of particular importance in threatened populations, which are often simultaneously subject to both inbreeding and environmental stress. But while the prevalence of inbreeding–stress interactions is well known, the importance and broader applicability of such interactions in conservation are not clearly understood. We used seed beetles, *Callosobruchus maculatus*, as a model system to quantify how environmental stressors (here host quality and temperature stress) interact with inbreeding as measured by changes in the magnitude of inbreeding depression, *δ*, as well as the relative importance of inbreeding–stress interactions to overall fitness. We found that while both environmental stressors caused substantial inbreeding–stress interactions as measured by change in *δ*, the relative importance of these interactions to overall survival was modest. This suggests that assessing inbreeding–stress interactions within the framework of *δ* alone may give an inaccurate representation of the relevance of interactions to population persistence. Furthermore, we found that the effect of environmental stress on fitness, but not inbreeding depression, varied strongly among populations. These results suggest that the outcomes of inbreeding–stress interactions are not easily generalized, an important consideration in conservation settings.

## 1 INTRODUCTION

It is now widely recognized that environmental stress can have a profound effect on the magnitude of inbreeding depression that a population experiences (Armbruster & Reed, 2005; Fox & Reed, 2011; Joubert & Bijlsma, 2010; Liao & Reed, 2009). But the relationship between environmental stress and inbreeding depression, while generally positive, has also been found to vary widely across systems (Armbruster & Reed, 2005). Many systems show a synergistic interaction between inbreeding depression and environmental stress, while others show no relationship, and still others suggest that environmental stress can actually decrease the magnitude of inbreeding depression (e.g., Armbruster & Reed, 2005; Dahlgaard & Hoffmann, 2000; Fox & Reed, 2011; Miller, 1994). For example, environmental stress appeared to decrease the magnitude of inbreeding depression in bladder campion plants and certain root parasites (Sandner & Matthies, 2016a, 2016b). This high degree of variation in the response to inbreeding–stress combinations suggests that inbreeding–environment interactions may not be easily generalizable across populations or species. Variable outcomes might be explained by differences in population history (e.g., purging history) or other causes (Hedrick & Garcia‐Dorado, 2016; Sandner & Matthies, 2016a). Knowing whether information regarding the effect of inbreeding–environment interactions in one threatened population can be dependably applied to other populations is of critical importance in the application of conservation policy. But the degree to which inbreeding depression varies across populations within a species or between closely related species remains relatively understudied (Fox, Scheibly, Smith, & Wallin, 2007).

Whereas inbreeding–stress interactions have been well‐studied within the framework of changes in inbreeding depression (i.e., studies assessing changes in the magnitude of inbreeding depression under benign vs. stressful conditions, see, e.g., Armbruster & Reed, 2005; Fox & Reed, 2011), studies comparing the relative importance of inbreeding–stress interactions versus additive effects on a population's overall fitness are less common. For threatened and endangered populations, which are likely to experience both inbreeding depression and environmental stress simultaneously, understanding the relative impact of inbreeding–stress interactions versus additive effects is of particular importance. If inbreeding–stress interactions have a far greater impact on fitness than the additive effects of individual stressors, failing to account for the interaction term could cause conservationists to underestimate a population's risk of extinction. Indeed, simulations conducted by Liao and Reed (2009) suggest that synergistic interactions between inbreeding and environmental stress could decrease the time for a population to go extinct by as much as 28.5%. In contrast, if the interaction term is negligible as compared to the additive effects of inbreeding or environmental stress, spending time and resources trying to minimize inbreeding–stress interactions could lead to a costly misallocation of conservation efforts. As such, understanding how inbreeding, environmental stressors, and their interactions compare in their relative effects on fitness—as well as how much this varies among populations or species—is crucial for conservation efforts (Armbruster & Reed, 2005; Kristensen, Barker, Pedersen, & Loeschcke, 2008; Kristensen, Dahlgaard, & Loeschcke, 2003; Pray, Schwartz, Goodnight, & Stevens, 1994; Reed, Briscoe, & Frankham, 2002; Reed, Fox, Enders, & Kristensen, 2012).

In this study, we used the cowpea seed beetle, *Callosobruchus maculatus*, as a model organism to assess the effect of two environmental stressors on both the magnitude of inbreeding depression and overall fitness across lineages. *Callosobruchus maculatus* is a widespread pest of stored legumes. Originally a pest of cowpea (*Vigna unguiculata*) in sub‐Saharan Africa, *C. maculatus* is now found in warm climates across the globe where it feeds on various species of grain legumes, especially from the tribe Phaseoleae (e.g., mung bean, adzuki bean, and cowpea; Kébé et al., 2017; Tuda, Rönn, Buranapanichpan, Wasano, & Arnqvist, 2006). Adults lay eggs on the surface of host seeds, and upon hatching, larvae burrow into a single seed where they remain for the entirety of their development. Under standard laboratory conditions, emerging adults do not feed, meaning beetles obtain all resources from a single seed. Thus, the “natural” habitat of *C. maculatus* populations infesting legume crop stores can be easily and precisely replicated under laboratory conditions (Messina, 1991; Tuda, Kagoshima, Toquenaga, & Arnqvist, 2014). Because larvae spend their entire life inside a single seed, host quality and temperature are the two primary environmental variables juvenile beetles experience. These life history characteristics make *C. maculatus* ideal for realistically manipulating ecological conditions.

Fox and Reed (2011) examined the degree to which inbreeding depression increases along two axes of stress (temperature and host) and across two populations of cowpea beetle (South India, SI, and Burkina Faso, BF). They found that inbreeding depression (as measured by haploid lethal equivalents, HLE) increased in a roughly linear fashion with the magnitude of environmental stress, but the overall effect of environmental stress on the magnitude of inbreeding depression varied across populations of *C. maculatus*. Specifically, they found that inbreeding depression in the BF lineage was less sensitive to changes in temperature stress than it was the SI lineage. We expand here on the work of Fox and Reed (2011) by using an additional cowpea beetle lineage to further our understanding of the generalizability of inbreeding–stress interactions. We also use a more stressful host species, green pea (average survival from hatched egg to adulthood on green pea, *Pisum sativum*, is around 30–50 percentage points lower than survival on either cowpea, *Vigna unguiculata*, or mung bean, *Vigna radiata*; see Messina, Lish, & Gompert, 2018), to more clearly assess two‐way versus three‐way interactions among stressors. Finally, we contrast the effect of inbreeding–stress interactions as measured by inbreeding depression with the effect of inbreeding–stress interactions on overall fitness. This dual perspective allows us to more clearly assess the conservation implications of interactions between environmental stress and inbreeding. In particular, we addressed the following questions: (a) To what degree do environmental stressors—and interactions among environmental stressors—affect the magnitude of inbreeding depression? (b) To what degree do individual stressors (inbreeding and environmental stress) versus interactions among those stressors impact overall fitness? And (c) to what degree do these effects vary by lineage?

## 2 METHODS

### 2.1 Experimental design

We used three lineages of *C. maculatus* for this experiment. The South India (SI) lineage was collected from mung bean (*V. radiata*) in Tirunelveli, India, in 1979 (Messina, 1991; Mitchell, 1991) and has been maintained in captivity in excess of 450 generations assuming an average generation time of 30 days. Two cowpea‐adapted lineages were obtained from Dr. Charles Fox at the University of Kentucky (Messina et al., 2018). Each was originally collected from infested cowpeas and has been maintained in the laboratory on this host continuously since their initial collection. The Burkina Faso (BF) lineage was collected from cowpea (*V. unguiculata*) pods in a field in Ouagadougou, Burkina Faso, in 1989 (Messina, 1993) and is estimated to have been maintained in captivity in excess of 325 generations. The North American lineage was collected from California (CA) and was originally maintained by Dr. Peter Credland at the University of London (Tuda et al., 2014). The CA lineage is estimated to have been maintained in laboratory culture for at least 100 generations. After all three lineages were obtained, cultures were maintained at Utah State University at 25°C in 2‐L jars containing 750 g beans for approximately 75 generations (BF and CA) or >100 generations (SI). New generations were founded approximately every 25 days by transferring ~2,000 (estimated by volume) newly emerged beetles to new 2‐L culture jars. Recent genomic analyses have shown that the mung‐adapted lineage (SI) used in this experiment has a variance effective population size of (*N*_e_) = 1,149.6 individuals (95% CI = 1,077.4‐1,229.8), indicating that significant bottlenecks or purging are unlikely to have occurred in the recent demographic history of this lineage (Gompert & Messina, 2016). The effective population sizes of CA and BF are expected to be similar or higher than that of SI given their extensive shared culturing history.

**FIGURE 1:**
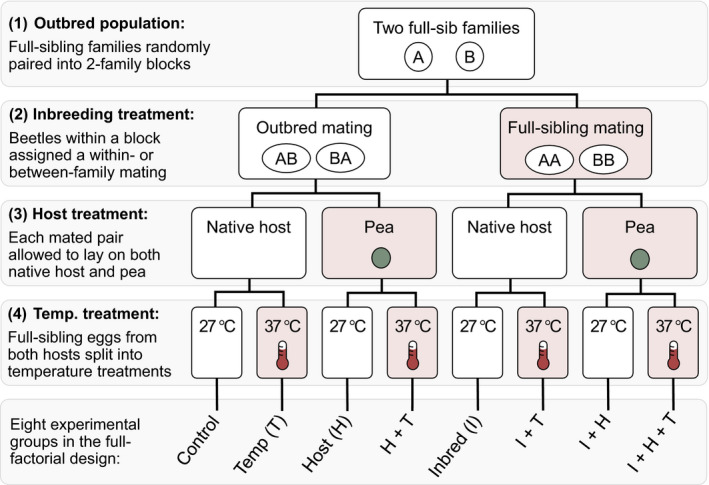
Flow chart showing how beetles were assigned to treatment groups within a block. We used a split‐brood design in order to divide the offspring of each beetle pair across all temperature–host treatment combinations. Red‐shaded boxes indicate stressful treatments, while white boxes indicate benign conditions. This experimental design was implemented for all blocks across all lineages (BF, CA, and SI)

We used a full‐factorial experimental design to assess the effect of two external stressors (host and temperature) and one internal stressor (inbreeding) on two fitness components (adult female weight and survival) across three populations of *C. maculatus* (SI, BF, and CA) (Figure 1). This design gives us eight distinct treatment groups: one control treatment, three single‐stress treatments, three double‐stress treatments, and one triple‐stress treatment. These eight combinations of stressors allow us to consider the importance of both two‐way and three‐way nonadditive effects of stressors on overall fitness.

To equalize the genetic contribution of all families to each treatment group and thereby control for family effects, we used the block design developed by Roff (1998) and used by Fox and Reed (2011). In this block design, full‐sibling offspring from two unrelated families are paired in four distinct crosses: two full‐sibling inbred crosses (one for each family) and two reciprocal outcrosses (i.e., a male from family A paired with a female from family B and a female from family A paired with a male from family B, see Figure 1). This design ensures equal contribution of alleles across treatment groups. Using reciprocal outcrosses helps account for family‐specific maternal/paternal effects. In addition, in this experiment we introduced a split‐brood design by dividing the eggs laid by each pair of beetles evenly across the environmental treatment groups, further controlling for family‐specific effects.

To create blocks, we first took 54 random pairs of virgin beetles from each population and allowed them to lay eggs. Single‐egg seeds from these founding pairs were then isolated in 48‐well tissue culture plates in order to obtain 54 full‐sibling families of up to 24 virgin beetles each. Successful full‐sibling families were randomly paired to form up to 26 blocks per population (where each block comprised two unique, unrelated full‐sibling families).

We conducted four types of crosses within each block: matings within each of the two full‐sibling families (inbreeding treatment, offspring inbreeding coefficient of *F* = 0.25) and reciprocal outcrosses between the two full‐sibling families (outbreeding treatment, *F* = 0) (Figure 1). We created up to five replicates of each cross type within each block (e.g., by conducting five full‐sibling crosses from ten members of a single full‐sibling family) to increase sample sizes and to account for within‐family variation in inbreeding depression.

We chose to use green pea, *Pisum sativum*, as the novel host for this experiment because peas impose a moderate level of stress as compared to the relatively high‐quality hosts (cowpea and mung bean) used in Fox and Reed (2011). Messina et al. (2018) found that in all cases, survival in green pea was well below the >90% survival seen in cowpea or mung, with survival on green pea ranging from ~40% to 72% dependent upon lineage. Thus, green pea can be viewed as not just a novel host, but a truly stressful host. All host seeds used in this experiment were organically grown and ordered in bulk to ensure uniform host quality. We acquired both cowpea and mung beans from Azure Standard (Dufur, OR) and green peas from Sun Organic Farm (San Marcos, CA).

All pairs of beetles from the above blocks were randomly assigned either their natural host (control; mung bean for SI, cowpea for BF and CA) or green pea (stressful host) as their first oviposition substrate. After 24 hr, if more than eight eggs had been laid by a given pair, the pair was transferred to a fresh petri dish with the alternate host. If fewer than eight eggs had been laid, the pair was left in the same petri dish for 1 additional day. Pairs were then transferred daily between the stressful host and the native host. This design was used to account for the possibility that (a) younger beetles lay more viable eggs (see, e.g., Fox, Bush, & Wallin, 2003; Fox & Reed, 2010), and (b) females may preferentially lay larger, or otherwise more fit, eggs on their native host. This split‐brood design allowed us to compare the effect of all experimental treatments within each full‐sibling family.

After 5–7 days, all seeds from each petri dish bearing a single egg were divided equally into two plastic bags, which were left partially unsealed to provide adequate air exchange, and placed into the heat stress treatment (37°C) or the control temperature treatment (27°C). Thus, the temperature exposure treatment in our study was from egg hatch to adult emergence. All beetles were reared in Percival incubators (Model Nos. AR‐22L and I‐36VL for heat and control temperature treatments, respectively) under a 12‐hr light:12‐hr dark cycle.

We measured performance in terms of survival (in all lines) and female adult mass (in BF and SI). Adult female mass was chosen as one of our fitness components because female size is often a good proxy for fecundity in insects, including in *Callosobruchus* (Credland, Dick, & Wright, 1986; Messina, 1993). After 15 days, bags were checked daily for emergence of adult beetles. Adult beetles were removed from the bags once every 24 hr and stored at −20°C in 48‐well tissue culture plates for subsequent mass measurements. Forty‐five days after the date the parental pairs (F1) were formed, the bags were removed from incubator and frozen to prevent the development of the next generation (F3). Development time for seed beetles at 25°C is generally <35 days on suitable hosts (Fox, 1993; Fox, Stillwell, Wallin, Curtis, & Reed, 2011; Messina, 1991; Messina & Durham, 2013). Thus, we measured survival for each treatment as emergence to 45 days. For any given bag, survival to adulthood was measured as the number of beans with exit holes divided by the total number of beans. We collected mass data from frozen female BF and CA beetles using a Mettler Toledo mass balance (model XPE105) with a precision of ±0.01 mg.

### 2.2 Statistical analysis

We fit Bayesian generalized linear models to quantify the individual and combined effects of inbreeding and the two environmental stressors (i.e., low‐quality host and high temperature) on *C. maculatus* survival and female mass (an approximation of fecundity). The output of a Bayesian model is a posterior probability distribution for model parameters of interest (in this case, the effect of environmental stressors, and the derived parameters, *δ* and HLE) based on experimental data, our mathematical model, and prior assumptions. To increase the efficiency of the computational model fitting process, we fit our model to each lineage separately. This is mathematically equivalent to fitting a single model for all three lineages and including population as a factor with a nonhierarchical prior (Kruschke, 2014). The resulting posteriors from these separate analyses can be directly compared and summarized across lineages, allowing us to make statistical inferences about differences among populations. Generating multiple summaries of a posterior distribution in a Bayesian analysis does not result in an increased risk of type I errors and is not subject to the problem of multiple testing as seen in conventional frequentist analysis (Kruschke, 2014).

### 2.3 Linear model

We assumed that the number of beetles that survived to the adult stage for each block (*j*) and treatment (*k*) was described by a binomial sampling distribution, that is, *y_jk_* ~ binomial (*p_jk_*, *n_jk_*), where *p_jk_* is the survival probability and *n_jk_* is the total sample size for the block and treatment. We further assumed that the logit probability of survival for block *j* and treatment *k* (denoted *p_jk_*) was a linear function of a block and treatment‐specific error term (*ϵ_j_*) and eight treatment covariates: an intercept, the three individual stress treatments (inbreeding, host, and temperature), three two‐way interactions (inbreeding × host, inbreeding × temperature, and host × temperature), and the single three‐way interaction (inbreeding × host × temperature), such that

$$\log\left(\frac{p_{\mathrm{jk}}}{1‐p_{\mathrm{jk}}}\right)\;=\;\beta^{C}\;+\;\beta^{I}1_{\mathrm{jk}}^{I}\;+\;\beta^{H}1_{\mathrm{jk}}^{H}\;+\;\beta^{T}1_{\mathrm{jk}}^{T}+\beta^{I\times H}1_{\mathrm{jk}}^{I\times H}+\beta^{I\times T}1_{\mathrm{jk}}^{I\times T}+\beta^{H\times T}1_{\mathrm{jk}}^{H\times T}+\beta^{I\times H\times T}1_{\mathrm{jk}}^{I\times H\times T}+\epsilon_{\mathrm{jk}}$$

Here, *I*, *H*, and *T* superscripts denote inbreeding, host, and temperature stress treatments, and the 1 are binary indicator variables set to 1 when all of the relevant stress treatments apply. We included the error terms (*ϵ_jk_*) to allow for over‐dispersion among blocks (i.e., treatment‐specific block effects) relative to simple binomial sampling expectations. Specifically, *ϵ_jk_* allows for a random effect for each block × treatment combination to account for the fact that individual pairings within a family block are not independent. We placed minimally informative priors on the eight regression coefficients, such that *β* ~ Normal(*μ* = 0, *τ* = 1e^−6^). Here, *τ* is the precision of the prior (*τ* = 1/*σ* ^2^). We modeled the *ϵ_jk_* terms hierarchically by assuming normal priors with means of zero and treatment‐specific precision parameters. Minimally informative priors were placed on the eight precision parameters, *τ_ϵ_* ~ gamma(0.1, 0.01).

We fit a similar model for the female mass data, but instead assumed a normal sampling distribution and the identity link function. We included the same eight covariates, that is, the intercept (*β^C^*), the three individual stress treatments (inbreeding, host, and temperature), three two‐way interactions (inbreeding × host, inbreeding × temperature, and host × temperature), and the single three‐way interaction (inbreeding × host × temperature). We likewise placed the same minimally informative normal priors on the regression coefficients (the *β* parameters) and assumed a minimally informative gamma prior for the precision parameter of the normal sampling distribution, that is, *τ* ~ gamma(0.1, 0.01). We estimated a separate precision parameter for each block and treatment, and included the random effect term *ϵ_jk_* for each block × treatment combination.

We fit the models via Markov chain Monte Carlo (MCMC) using the rjags (version 4‐8) interface with JAGS (version 4.2.0) (Plummer, 2003, 2013). For each model (i.e., lineage and performance metric), we ran three chains, each with a burn‐in period of 2,000 iterations, a thinning interval of 100 (survival) or 50 (mass), and 200,000 (survival) or 50,000 (mass) post‐burn‐in iterations. We evaluated convergence to the posterior distribution by examining sample history plots and by calculating both parameter effective sample sizes and the Gelman–Rubin scale reduction factor (Gelman & Rubin, 1992).

We focused our inferences on Bayesian point estimates (posterior medians) and 95% credible intervals (CIs, specifically, equal‐tail probability intervals) of the regression coefficients and several derived parameters. Specifically, from the posterior samples, we calculated posterior probability distributions for expected survival probabilities and mass for each treatment (across blocks) and differences in expectations between treatments.

### 2.4 Calculation of inbreeding depression

Within each model, we also calculated the coefficient of inbreeding depression from the posterior distributions for each of the four inbred–outbred treatment pairs: outbred versus inbred, outbred‐host stress versus inbred‐host stress, outbred‐temperature stress versus inbred‐temperature stress, and outbred‐host and temperature stress versus inbred‐host and temperature stress. The coefficient of inbreeding depression, *δ*, is defined as

(1) $$\delta \;=\;\frac{W_{o}‐W_{i}}{W_{o}}$$

where *W*_o_ and *W_i_* denote relative fitness of outbred (o) and inbred (*i*) lines, and *δ* thus represents the percent change in fitness attributable to inbreeding. *δ* is bounded between zero and 1, where 1 represents a 100% decline in fitness (i.e., survival or mass) due to inbreeding and 0 represents the case where no inbreeding depression occurred. In the context of our Bayesian model, *δ* was calculated as a derived parameter by subtracting the posterior samples (MCMC output) for fitness in the inbred group from the posterior samples for fitness in the outbred group, then dividing the result by posterior samples for fitness in the outbred group. The output of this calculation is a posterior distribution for *δ* for each environmental treatment group. We likewise calculated the number of haploid lethal equivalents (HLE) over the posterior for each outbred versus inbred treatment comparison as

(2) $$\text{HLE}\;=\;\frac{\log\left(\frac{W_{i}}{W_{o}}\right)}{0.25}$$

Here, 0.25 denotes the inbreeding coefficient, *F*, or the probability that an individual received two identical copies of an allele from the same ancestor. Because our inbreeding treatment included solely the offspring of full‐sibling matings, all beetles in our inbred treatment groups will have an inbreeding coefficient of *F* = 0.25. As with *δ*, HLE was calculated as a derived parameter using the posterior distributions from our Bayesian linear models. Haploid lethal equivalents (HLEs) can be interpreted as the number of lethal loci required to produce the observed drop in fitness associated with inbreeding in a haploid population. Thus, if HLE = 4, it would indicate that this population carries the equivalent of four lethal alleles, though in reality the population may carry a different number of deleterious alleles of lesser effect. Finally, we calculated the effect of environmental stress on the magnitude of inbreeding depression as the posterior differences between *δ* ^stress^ and *δ* ^control^ (i.e., *δ^T^* − *δ^C^* and *δ^H^* − *δ^C^*), and the two‐way interaction between heat and temperature stress on inbreeding depression as *δ^H^* ^+^ *^T^* − *δ^H^* − *δ^T^* + *δ^C^*.

## 3 RESULTS

We mated 200, 254, and 358 pairs of virgin beetles each from the CA, BF, and SI lineages, respectively, to produce a total of 17, 20, and 23 complete blocks. A block was considered complete if each of the four distinct cross types (inbred pairs from two families and their reciprocal outcrosses) was represented by at least one replicate pair within that block. From these pairs, we collected a total of 31,239 single‐egg seeds (CA = 7,464 beans, BF = 10,077 beans, SI = 13,698 beans). After excluding data showing evidence of F1 beetles having mated and produced a second generation, we were left with survival data for 30,746 single‐egg seeds (CA = 7,316 beans, BF = 9,932 beans, SI = 13,498 beans), which we used in our analysis. On average, each treatment group contained survival data from 1,281 beans.

### 3.1 Survival

**FIGURE 2:**
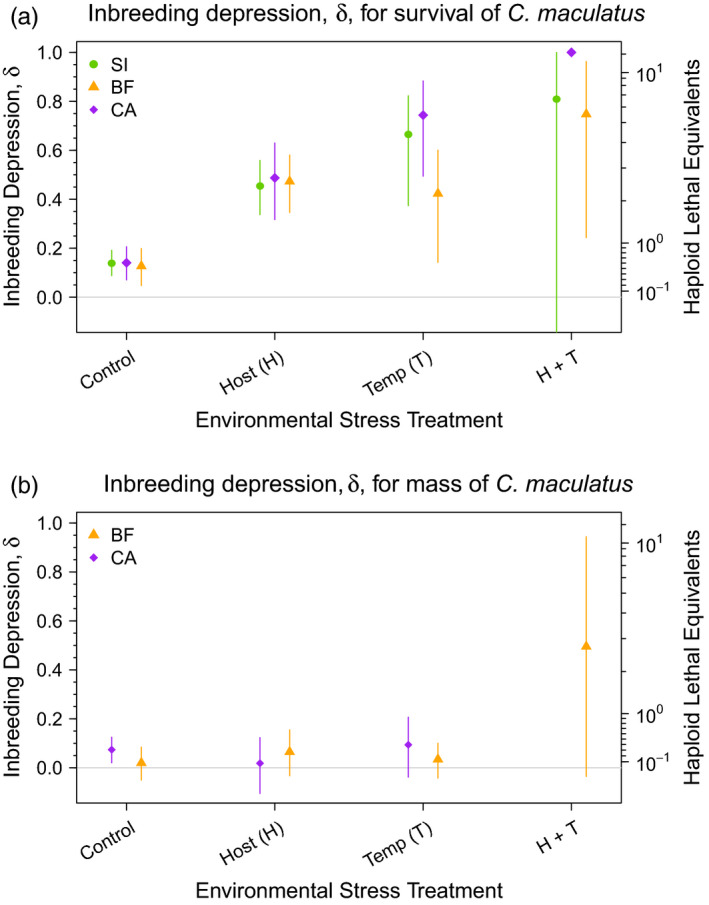
Model posterior summaries for inbreeding depression (*δ*) and haploid lethal equivalents (HLE) using (a) survival and (b) mass as the measure of fitness. Points represent the median value of the posterior for each treatment–lineage combination, while vertical bars denote the 95% credible interval. Values of *δ* range from 0 to 1, with zero indicating no inbreeding depression and 1 indicating that inbreeding reduced fitness by 100%. HLE is represented on a log scale, with the power of 10 increasing by 0.1 with each hash mark

Host stress, temperature stress, and the combination of both all substantially increased the magnitude of inbreeding depression (as captured by *δ*) for survival in *C. maculatus* (posterior probabilities [p.p.] *δ^H^* > *δ* ^control^ ≥ 0.99, 0.99, 0.99; p.p. *δ^T^* > *δ* ^control^ = 0.99, 0.99, 0.98; p.p. *δ^H^* ^+^ *^T^* > *δ* ^control^ = 0.91, 0.99, 0.99 for SI, CA, and BF lineages, respectively) (see Table 1). In the benign environment, inbreeding depression decreased survival by 12.7%–14.0%, while under host stress it decreased survival by 45.4%–48.7% across lineages (see Figure 2a). Temperature stress had a more variable effect on inbreeding depression, decreasing survival by 42.3%, 66.6%, and 74.4% for BF, SI, and CA lineages, respectively. Finally, inbreeding depression decreased survival by 74.8%–100% percent across lineages when both environmental stressors (temperature and host) were present (Figure 2a). Analogous results in terms of haploid lethal equivalents (HLE) are shown in Figure 2a.

**TABLE 1:** Point estimates and 95% credible intervals for the effect of host stress, temperature stress, and host–temperature stress interactions (*H* × *T*) on inbreeding depression, *δ*, using both survival and mass and fitness measures

	Host (*δ^H^* − *δ^C^*)	Temp (*δ^T^* − *δ^C^*)	*H* × *T*
Survival			
SI	0.32 (0.19, 0.43)	0.53 (0.23, 0.70)	−0.18 (−1.77, 0.21)
BF	0.35 (0.20, 0.48)	0.30 (0.01, 0.49)	−0.03 (−0.58, 0.38)
CA	0.35 (0.16, 0.51)	0.60 (0.35, 0.76)	−0.08 (−0.31, 0.21)
Mass			
BF	0.04 (−0.07, 0.16)	0.01 (−0.09, 0.11)	0.42 (−0.14, 0.88)
CA	−0.06 (−0.19, 0.06)	0.02 (−0.12, 0.15)	–

Note

Values greater than zero represent treatments or interactions that increased the severity of inbreeding depression, while values less than zero represent those that decreased the severity of inbreeding depression. Host–temperature interactions were calculated as *δ^H^* ^×^ *^T^* = *δ^H^* ^+^ *^T^*−*δ^H^* − *δ^T^*+*δ^C^*.

Although inbreeding depression increased substantially under the combination of temperature and host stress relative to the control (*δ^H^* ^+^ *^T^* was 5.6×, 7.1×, and 5.7× greater than *δ* ^control^ for SI, CA, and BF, respectively), the magnitude of this increase was equal or less than additive relative to the effects of the individual stressors. Specifically, we saw a modest trend toward a negative interaction between host and temperature stress on inbreeding depression (p.p. *δ^H^* ^×^ *^T^* < 0 = 0.81, 0.73, and 0.55 for SI, CA, and BF, respectively, see Table 1). In other words, the combination of host and temperature stress on inbreeding depression had an effect equal to or less severe than the sum of their separate effects.

**FIGURE 3:**
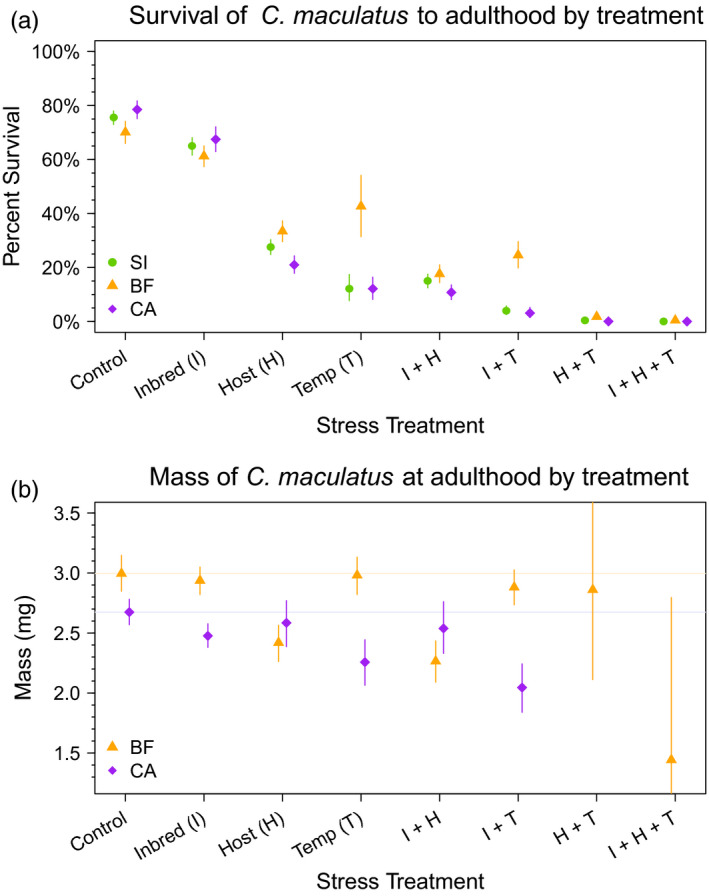
Model posterior summaries for (a) percent survival and (b) female mass by treatment group and lineage. Points represent the median value of the posterior for a given treatment–lineage combination, and vertical bars denote the 95% credible interval. Horizontal lines in (b) represent the median mass for the control treatment by lineage

**FIGURE 4:**
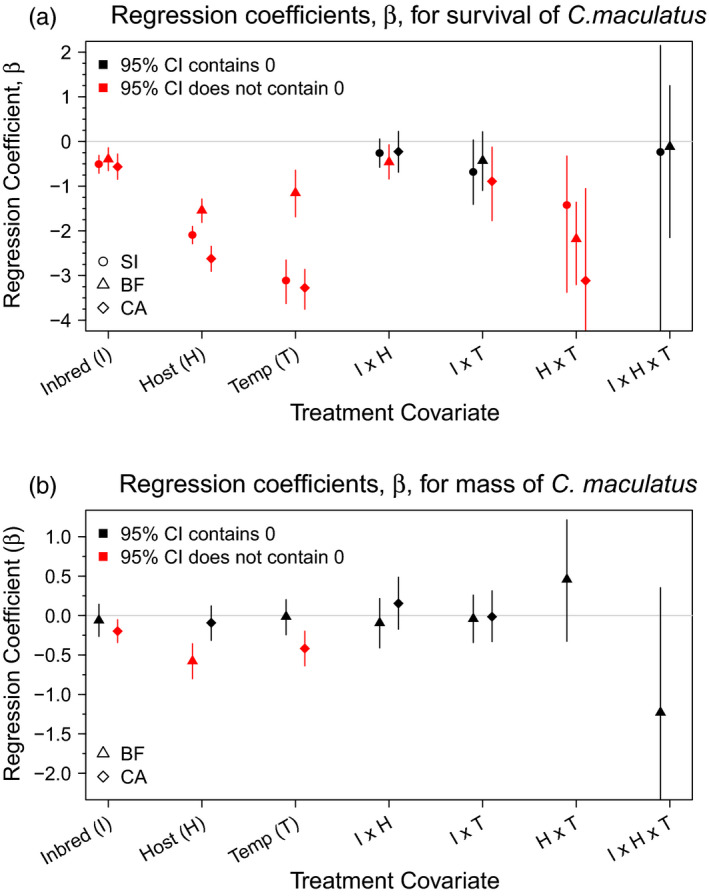
Model posterior summaries for regression coefficients (*β*) for each of the seven treatment covariates from the linear models for (a) survival and (b) mass. Regression coefficients are presented on a logit scale where points indicate the median value of *β* from the posterior and vertical bars indicate 95% credible intervals. The horizontal gray bar indicates the line *y* = 0. Regression coefficients falling below zero indicate variables which decreased survival or mass as compared to the control, while those above zero increased survival or mass

All stress treatments—inbreeding, host, temperature, and every combination thereof—decreased *C. maculatus* survival to adulthood (p.p. for reduced survival relative to the control >0.99 for all stress treatments) (Figures 3a and 4a). Across lineages, survival to adulthood under host stress was 36.7–57.5 percentage points lower than under benign conditions, while under temperature stress survival decreased by 27.5–66.3 percentage points (see Figure 3a). Inbreeding depression had less of an impact on survival (8.9–11.0 percentage point decrease) than either host or temperature stress, and showed less variation across lineages. Across all lineages, adding inbreeding stress to environmental stress lowered survival by an additional 8.1–18.1 percentage points compared to the environmental stress treatment alone (compare *I* + *H* to *H* and *I* + *T* to *T* in Figure 3a). Finally, both the combination of temperature and host stress and the combination of all three stressors (inbreeding, temperature, and host) imposed such severe stress that survival dropped to <2% across lineages.

In contrast to our results for inbreeding depression (*δ*), the reduction in fitness under combinations of two stressors showed a trend for being greater than additive (p.p. for *β_I_*_×_ *_H_* < 0 = 0.84–0.99, *β_I_*_×_ *_T_* < 0 = 0.90–0.99, *β_H_*_×_ *_T_* < 0 > 0.99) (Figure 4a). However, the only combination of stressors that showed a consistent nonadditive impact on survival to adulthood was host × temperature (see Figure 4a). While the 95% CIs for the host × inbreeding and temperature × inbreeding treatments overlapped zero for most lineages, there were credible interactions between host and inbreeding in BF and between temperature and inbreeding in CA (p.p. *β_I_*_×_ *_H_* < 0 = 0.99 for BF and p.p. *β_I_*_×_ *_T_* < 0 = 0.99 for CA). Three‐way interactions showed considerable uncertainty and were not credibly different from zero.

Finally, there were no credible differences in the magnitude of inbreeding depression across any of our populations (see Table 2). In contrast, the effect of host stress varied credibly across all three populations (Table 2). In particular, both temperature and host stress had a credibly lower impact on survival in BF than in either SI or CA (Table 2). This trend was particularly pronounced for temperature stress, where CA and SI survival was 30 percentage points lower than in BF (95% CI = 0.18–0.43). Moreover, survival in BF was affected more by host than temperature (*β_H_* − *β_T_* = −0.39, 95% CI = −0.90 to 0.13, p.p. < 0 = 0.93), while survival in CA and SI was affected more by temperature than host (CA, *β_H_* − *β_T_* = 0.65, 95% CI = 0.23–1.13, p.p. > 0 > 0.99; SI, *β_H_* − *β_T_* = 1.01, 95% CI = 0.56–1.53, p.p. > 0 > 0.99).

**TABLE 2:** Point estimates and 95% credible intervals for differences between populations by treatment group for both survival and mass data

	Control	Inbred	Host	Temp
Survival				
BF‐CA	**−0.08 (−0.14, −0.03)**	−0.06 (−0.12, 0.00)	**0.13 (0.07, 0.18)**	**0.30 (0.19, 0.43)**
BF‐SI	**−0.05 (−0.10, −0.01)**	−0.04 (−0.09, 0.01)	**0.06 (0.01, 0.11)**	**0.30 (0.18, 0.43)**
CA‐SI	0.03 (−0.01, 0.07)	0.02 (−0.03, 0.08)	**−0.07 (−0.10, −0.02)**	0.00 (−0.07, 0.06)
Mass				
BF‐CA	**0.32 (0.14, 0.51)**	**0.46 (0.31, 0.61)**	−0.16 (−0.40, 0.07)	**0.73 (0.48, 0.97)**

	*I* + *H*	*I* + *T*	*H* + *T*	*I* + *H* + *T*
Survival				
BF‐CA	**0.07 (0.03, 0.11)**	**0.21 (0.16, 0.27)**	**0.02 (0.01, 0.03)**	0.00 (0.00, 0.01)
BF‐SI	0.03 (−0.02, 0.07)	**0.21 (0.15, 0.26)**	0.01 (0.00, 0.03)	0.00 (0.00, 0.01)
CA‐SI	**−0.04 (−0.08, −0.01)**	−0.01 (−0.03, 0.02)	0.00 ( −0.01, 0.00)	0.00 (0.00, 0.00)
Mass				
BF‐CA	**−0.28 (−0.55, −0.01)**	**0.84 (0.59, 1.09)**	–	–

Note

Differences between population pairs were calculated as derived parameters by taking the difference between the posterior samples for the first population and the second, then summarizing the output. Negative estimates indicate that the second population had a higher survival or mass value than the first, while positive estimates indicate that the second population had a lower survival or mass value than the first. Credible differences between populations (in either direction) are shown in bold.

Similarly, in the double‐stress (*I* + *H*, *I* + *T*, and *H* + *T*) treatments, the CA and SI lineages showed credibly lower survival than BF (a 21 percentage point decrease; 95% CI = 0.15–1.27, see Table 2). The effect of host stress on survival was also credibly different across all population pairs (p.p. that the absolute value of the difference in *β^H^* ^^> 0 ≥ 0.99 for all three population pairs). The effect of temperature stress was credibly less severe than in either CA or BF (p.p. that $\beta_{\mathrm{BF}}^{T}‐\beta_{\mathrm{CA}}^{H}$ and $\beta_{\mathrm{BF}}^{T}‐\beta_{\mathrm{SI}}^{H}$ > 0 ≥ 0.99). However, no credible differences between populations were observed for interaction terms (i.e., *β_I_*_×_ *_H_*, *β_I_*_×_ *_T_*, and *β_H_*_×_ *_T_*), indicating that the differences across populations in our double‐stress groups are largely due to differences in the additive effects of each stressor across populations rather than differences in the magnitude of interactions across populations. No credible differences between populations were observed in the triple‐stress (inbreeding + host + temperature) treatment or for the three‐way interaction term (*β_I_*_×_ *_H_*_×_ *_T_*). Similar results were observed for the effects of external stressors on inbreeding depression (*δ*) (Figure 2a).

### 3.2 Female mass

Under benign conditions, we saw inbreeding depression with respect to female mass only in the CA lineage, where inbreeding decreased mass by 7.4% (95% CI = 2.0–12.5, see Figure 3b). In the single‐stress treatments, we saw a trend for inbreeding depression under host stress in BF (p.p. *δ* ^host^ > 0 = 0.901), and under temperature stress in CA (p.p. *δ* ^temp^ > 0 = 0.929). However, evidence that either of these treatments increased inbreeding depression more than in the control treatment was moderate to marginal (p.p. *δ* ^host^ > *δ* ^control^ = 0.770 for BF; p.p. *δ* ^temp^ > *δ* ^control^ = 0.615 for CA, see Table 1). Analogous results in terms of haploid lethal equivalents (HLE) are shown in Figure 2b.

We were unable to obtain female mass data for the CA host–temperature combination stress treatment because survival was too low. However, the two‐way combination of temperature and host stress showed a strong trend for increasing the magnitude of inbreeding depression in BF (Figure 2b). Inbred BF beetles from the temperature plus host treatments were 49.2% (95% CI = −16.5 to –94.8) smaller by mass than noninbred BF beetles subjected to those same environmental stressors (as compared to 2.02%, 6.53%, and 3.24% for *δ* ^control^, *δ* ^host^, and *δ* ^temp^, respectively). Furthermore, the trend for the magnitude of this increase in inbreeding depression was more than additive: Inbreeding depression increased by 41.7 percentage points more than expected by simply summing the effects of temperature and host stress (95% CI = −13.7 to 87.8 percentage points; p.p. *δ^H^* ^+^ *^T^* > *δ^H^* + *δ^T^* − *δ^C^* = 0.96). In contrast to our results for survival (where we saw a modest negative trend), here the trend for an interaction between host and temperature stress on inbreeding depression was strongly positive (Figure 2b and Table 1).

There was a highly credible difference in female mass across lineages: Under benign conditions, average mass for the BF lineage was 11% greater than for CA (CA, mean mass = 2.67 mg, 95% CI 2.57–2.78 mg; BF, mean mass = 3.00 mg, 95% CI 2.85–3.14 mg for BF, see Figure 3b and Table 2). While inbreeding decreased female mass in both lineages, only CA showed a credible effect (BF, p.p. *β^I^* < 0 = 0.72; CA, p.p. *β^I^* < 0 ≥ 0.99) (Figures 3b and 4b). As with survival, there were no credible differences in the effect of inbreeding depression (*β^I^*) on female mass across populations. However, the effect of host stress on female mass was considerably more severe in BF than in CA (p.p. *β^H^* for BF < *β^H^* for CA ≥ 0.99). In the BF lineage, host stress decreased female mass at adulthood by 0.578 mg (95% CI = 0.357 to 0.801 mg decrease) as compared to the control group. While host stress showed no measurable effect on mass in CA, temperature stress decreased CA mass from 2.67 to 2.26 mg (a 0.41 mg drop; p.p. mass^temp^ > mass^control^ > 0.99) (Figure 3b). In contrast, the effect of temperature stress on CA was considerably more severe in CA than in BF (p.p. *β^T^* for CA < *β^T^* for BF = 0.99, see Figure 4b).

We found no credible evidence of two‐way interactions between stressors on mass in either BF or CA (Figure 4b), and we saw no credible differences in the interaction terms (*β^I^* ^×^ *^H^* and *β^I^* ^×^ *^T^*) across populations. As for survival, this indicates that the credible differences in female mass we saw in the double‐stress treatment groups (i.e., *I* + *H* and *I* + *T*, see Table 2) can be attributed mainly differences in baseline female mass and the differential effects of additive stressors across populations rather than differences in the magnitude of interactions across populations. That said, we did find a strong positive trend for an interaction among all three stressors combined (inbreeding, host, and temperature stress) on mass in BF (p.p. *β^I^* ^×^ *^H^* ^×^ *^T^* < 0 = 0.96), such that beetles exposed to all three stressors were smaller than expected from the effects of the individual stressors. Due to exceedingly low survival in the host plus temperature treatment and in the inbreeding plus host plus temperature treatments, we were unable to collect mass data for CA (Table 3).

**TABLE 3:** Mass sample sizes by lineage and treatment

	Control	Inbred (*I*)	Host (*H*)	Temp (*T*)	*I* + *H*	*I* + *T*	*H* + *T*	*I* + *H* + *T*
BF	371	349	200	241	94	139	9	3
CA	291	237	71	49	49	19	0	0

## 4 DISCUSSION

The prevalence of interactions between inbreeding and environmental stress has become clear in recent years, garnering interest in what consequences this might hold for conservation (Armbruster & Reed, 2005; Fox & Reed, 2011; Kristensen et al., 2003, 2008; Liao & Reed, 2009; Pray et al., 1994; Reed et al., 2012). But the significance and consistency of this phenomenon remain unclear. In this study, we sought to shed light on these questions by approaching analyses from two different perspectives: First, we looked at the degree to which environmental stress (and interactions among stressors) resulted in inbreeding–stress interactions as measured by the magnitude of inbreeding depression (*δ*). Second, we looked at the degree to which interactions among each combination of our three stressors (inbreeding, host, and temperature) impacted overall fitness, as measured by survival and female mass. By comparing the effect of inbreeding–stress interactions as measured by the effect of environmental stressors on inbreeding depression versus the effect of inbreeding–stress interactions on fitness, we sought to determine whether a substantial inbreeding–stress interaction as measured by *δ* implies that the effect of that interaction on overall fitness will be relevant for conservation. We used *C. maculatus* lineages from three different continents (CA from North America, BF from Africa, and SI from southwest Asia) to determine the degree of consistency in stress responses across lineages.

### 4.1 Inbreeding depression and overall fitness are not interchangeable ways to measure inbreeding‐stress interactions

Both environmental stressors we used (host and temperature stress) had a strong effect on the magnitude of inbreeding depression, *δ*, for survival in our lineages. This is consistent with the general literature consensus that environmental stress generally increases the severity of inbreeding depression (Armbruster & Reed, 2005; Fox & Reed, 2011). Similarly, we found that each stressor individually (inbreeding, temperature, and host) had a strong impact on *C. maculatus* survival. Our environmental stressors (temperature and poor host quality) caused a greater reduction in survival in *C. maculatus* than did inbreeding. That said, under our experimental design beetles were subjected to only one generation of inbreeding (inbreeding coefficient *F* = 0.25). In the wild (or in captive breeding), populations may instead be subjected to prolonged bouts of inbreeding. Thus, the current study may underestimate the impact of inbreeding depression in wild or captive‐bred populations. Conversely, in cases where inbreeding has been so prolonged that purging has occurred (see Bijlsma, Bundgaard, & Van Putten, 1999), our study may instead overestimate the impact of inbreeding depression.

Despite finding clear evidence that both environmental stressors produced an inbreeding–stress interaction as measured by their effect on *δ*, the survival model showed an inbreeding–stress interaction in only two treatment groups: inbreeding × host in BF and inbreeding × temperature in CA. This discrepancy illustrates that inbreeding–stress interactions showing credible effects within the context of relative fitness (i.e., outbred vs. inbred fitness) may show marginal effects within the context of overall fitness. Instead, the decline in survival in our treatment groups can be attributed largely to the individual, additive effects of each stressor rather than inbreeding–stress interactions. Thus, even in cases where the effect of an inbreeding–stress interaction as measured by inbreeding depression (i.e., *δ* or HLE) is substantial, we cannot assume that the importance of those interactions versus additive effects of individual stressors on overall fitness will be similarly important. For example, if under benign conditions, inbreeding decreased survival from 100% to 90%, while under stressful conditions, inbreeding decreased survival from 2% to 1%, *δ* would increase from 0.1 to 0.5, indicating that a substantial inbreeding–stress interaction exists. But concluding from this change in *δ* that inbreeding–stress interactions have a substantial effect on population fitness would be false, as in this hypothetical scenario the additive effects of environmental stress alone caused a 98% drop in survival, rendering the effect of the inbreeding–stress interaction negligible by comparison. Thus, using *δ* as the sole measure of the importance of inbreeding–stress interactions may give a false impression regarding the relative importance of inbreeding–stress interactions to overall fitness. Our findings highlight the importance of carefully parsing the effect of inbreeding–stress interactions as measured by inbreeding depression versus the effect of inbreeding–stress interactions as measured by overall fitness—relevance in the first case may not imply relevance in the second.

### 4.2 Strength of interactions depends on the severity of the stressors

Because our study expands upon the work of Fox and Reed (2011), some of our results can be compared. Overall, Fox and Reed (2011) saw higher baseline survival and more severe inbreeding depression than we did (*δ* ^control^ = 0.31 for BF and 0.19 for SI, as compared to 0.13 and 0.14 in our study). We also saw a drastically different response to temperature stress in the SI lineage. Whereas Fox and Reed (2011) found that temperature stress decreased survival in SI by only 13 percentage points (88% survival in the control vs. 75% under temperature stress), we found that temperature stress decreased survival in SI by 63 percentage points (75.5% survival in the control vs. 12.1% under temperature stress). These differences may reflect the impact of confounding variables such as humidity, host seed quality, differences in rearing setup, and oxygen availability. Alternatively, there may have been significant changes in the genetic composition of the SI population between the earlier study and ours. Under most temperature regimes, seed beetles have a generation time of approximately 30 days or less (Fox et al., 2011). Consequently, the SI and BF lineages would have had over 75 generations in which to evolve differences between the Fox and Reed (2011) experiment and our own. Fricke and Arnqvist (2004) demonstrated that replicate populations of the same seed‐beetle strain can rapidly diverge in mating behavior and reproduction under different laboratory conditions. Both local adaptation and genetic drift could cause substantial change in a population's response to both environmental stress and inbreeding. Accounting for the potential of evolutionary rescue may an important consideration to avoid over‐generalizing the effect of inbreeding–stress interactions both across populations and over time (Carlson, Cunningham, & Westley, 2014; Gonzalez, Ronce, Ferriere, & Hochberg, 2013).

The consensus of our study, Fox et al. (2011), and Fox and Reed (2011) suggest that the severity of an individual stressor may be critical to determining its potential for interactions with other stressors or with inbreeding. Fox et al. (2011) used mild stressors and found marginal evidence for interactions among stressors. The authors suggested that the range of stressors used were so mild that either (a) the interactions between stressors were too small to detect, or (b) stressors must reach a certain severity threshold before interactions occur between them. Fox and Reed (2011) used moderate stressors (e.g., survival in the most stressful treatment group was 33%) and found clear evidence for relevant interactions among them. In this study, we used combinations of stressors that in some cases severely limited survival, with three treatment groups showing survival of <2% (*I* + *T*, *H* + *T*, and *I* + *H* + *T*). Thus, our study expands upon Fox et al. (2011) and Fox and Reed (2011) to represent the far end of the spectrum: the effect of interactions when stressors are severe. In our most severe stress combinations, we found little evidence of interactions. This result provides strong support for the trend that Schou, Loeschcke, and Kristensen (2015) found in their study of inbreeding–stress interactions in *Drosophila*: Under severe stress, the rate of increase in inbreeding–stress interactions falls short of linear. In other words, the higher the stress level is, the smaller the increase in inbreeding–stress interactions becomes.

Taken together, these trends illustrate that although the strength of interactions may increase with the severity of stress imposed when stressors are mild to moderate, there will be a point at which the interaction between two biological stressors will necessarily be less than additive due to limiting bounds on the values a trait can assume. For example, inbreeding depression, *δ*, has a maximum upper bound of 1, which would indicate that inbreeding decreased fitness by 100%. Consider inbreeding depression for survival in the CA lineage in our study: Under a purely additive model, we would predict that *δ^H^* ^+^ *^T^* should decrease survival by 108.8% (baseline *δ* plus additional effect of temperature and host = 14% + 60.1% + 34.7%, see Table 1), which of course is impossible. Thus, when assessing the relevance of potential interactions in a conservation context, it may be helpful to first consider the magnitude of each stressor on its own.

### 4.3 Interactions vary with the fitness component measured

Our study also showed that effects of inbreeding and stress depend on fitness measure. We saw clear evidence of inbreeding depression and the impact of individual stressors on *δ* for survival, but these effects were less clear for adult female mass. The effect of interactions between environmental stressors also varied by fitness component. Using female mass as our measure of fitness, we found a strong trend for a positive interaction between host and temperature stress on inbreeding depression. This is consistent with Fox and Reed (2011) and with studies showing increased inbreeding depression under stressful conditions (e.g., Armbruster & Reed, 2005; Liao & Reed, 2009). But we saw precisely the opposite when survival was the fitness measure: The interaction between temperature and host was negative (see Figure 2). Hence, for survival, the effect of temperature plus host stress was less severe than the sum of their separate effects. Similarly, we did not see evidence for a three‐way inbreeding × host × temperature interaction for survival, but there was a strong trend for a three‐way interaction for mass.

The consistent differences between our survival versus mass results underscore the fact that not all components of fitness will respond the same way to inbreeding or environmental stress. This point was noted previously by Armbruster and Reed (2005) and Charlesworth and Charlesworth (1987) in their reviews of inbreeding literature. In our case, the differences in the stress response of mass versus survival may have arisen in part because beetles with low mass were less likely to survive to adult emergence. Thus, the mean weight of beetles that emerged successfully may be higher than would have been observed had all beetles survived to emergence. In other cases, such as for the three‐way stress interaction on survival, the lack of effect might have occurred because the two‐way stress treatment (temperature + host) alone decreased survival to <2%, a value too low to allow us to detect a more‐than‐additive three‐way interaction. We note that our results are closely parallel to those of Schou et al. (2015), who also found that the magnitude of inbreeding depression was lower when measured using mass rather than survival in *Drosophila*, suggesting that this trend may be generalizable in certain cases. These results also reinforce the notion that fitness components are not always directly comparable, and it would be advisable to avoid generalizing the results of a single fitness component. In addition, our results suggest that it is important to think carefully about which fitness measure (or combination of fitness components) is most relevant for the particular organism under study.

### 4.4 Effect of environmental stress differs across lineages

We found little evidence for variation in the magnitude of inbreeding depression among lineages. In wild populations that have undergone drastic bottlenecks or other significant demographic changes (as might be expected in endangered populations), population‐specific responses may be more common. As the variance effective population size (*N*_e_) for the least‐fecund lineage in our experiment was equal to *N*_e_ = 1,149.6, it is unlikely that drastic bottlenecks have occurred in our beetle lineages' recent history (Gompert & Messina, 2016). That said, the beetle lineages we used have been maintained in captivity for in excess of 100 generations (and over 450 generations in the case of SI), meaning that substantial levels of genetic drift and adaptation to captivity are likely to have occurred in our lineages.

We did observe, however, that the response to environmental stressors, but not their interactions with inbreeding depression, varied strongly across populations. Specifically, we found that the Burkina Faso (BF) lineage was less susceptible to environmental stress overall and in particular showed far greater tolerance to temperature stress. In contrast, the South India (SI) and California (CA) populations were more susceptible to temperature stress than to host stress. In addition to differences in the mean effect of environmental stress, the variance in response to environmental stress seen in BF was also far higher than in the South India (SI) and California (CA) populations (see Figure 3a). While we saw no credible differences in inbreeding–stress interactions across populations, we did see a modest trend for the host × temperature interaction being greater in BF than CA (see Figure 4a). Differences in response to temperature stress across populations (both mean and variance) may reflect local adaptation in each lineage to different stressors in their native environments, but given the differences in results between Fox and Reed (2011) and our study, it seems likely that divergent assay conditions and prior laboratory evolution (adaptation to captivity and/or drift) may have played a role. Nevertheless, these differences suggest that the effect of environmental stress may not generalize well across populations. Inbreeding–stress interactions, however, were more consistent, suggesting that the magnitude of inbreeding–stress interactions may be somewhat more generalizable across populations within a single species than are the effects of individual stressors.

### 4.5 Conservation implications and future directions

In conclusion, we found that the magnitude and relevance of individual stressors and their interactions varied with (a) analysis perspective (i.e., measuring inbreeding–stress interactions in the context of inbreeding depression vs. fitness) (b) fitness component, (c) stressor severity, and (d) population. In all, our results suggest that inbreeding–stress interactions are both variable and complex. Critically assessing the aforementioned factors may help better clarify under which circumstances inbreeding–stress interactions are relevant for applied conservation. In particular, our results suggest that *δ* or HLE may be more sensitive measures for assessing the presence of inbreeding–stress interactions as they are based on relative rather than absolute fitness (specifically, inbred relative to outbred fitness). Thus, in situations where the goal is to determine the presence of an inbreeding–stress interaction, no matter how small, *δ* or HLE may prove more effective. However, in the context of conservation, it is arguably more important to understand not whether an inbreeding–stress interaction exists, but whether an interaction's effects are large enough to warrant conservation concern. Measures such as *δ* and HLE are not necessarily the most effective way to determine the relative importance of interactions versus the additive effects of stressors. Thus, we suggest that placing inbreeding–stress interactions within the context of overall fitness (i.e., comparing additive vs. interactive effects directly) will yield more informative results regarding the conservation relevance of inbreeding–stress interactions than will looking at inbreeding–stress interactions in terms of *δ* or HLE alone.

In addition to exercising caution when interpreting the relevance of inbreeding–stress interactions within the framework of *δ* alone, our study showed that the magnitude of inbreeding–stress interactions varied with both fitness component and degree of stress imposed. This is consistent with previous research (Armbruster & Reed, 2005; Charlesworth & Charlesworth, 1987; Schou et al., 2015) and underscores the inbreeding–stress interactions can vary widely depending on the severity of stress a population experiences and which fitness measure is used. Future studies exploring the relationship between the magnitude of stressors and the resulting interaction between them could help explain some of this variation and shed further light on how broadly inbreeding–stress interactions can be generalized. For conservation purposes, fitness measures of direct relevance to population persistence or management (in particular survival and fecundity) may be of greater value than fitness measures with less obvious connections to population persistence.

Finally, investigating the role of local adaptation and population demographic history (in particular the effects of small population size or bottlenecks) may help us better explain and predict variation in inbreeding–stress interactions across populations. While we know that inbreeding–stress interactions are a widespread phenomenon, how such interactions may change under adaptive evolution, evolutionary rescue, or purging is unclear. While our study did not show substantial differences in inbreeding depression or inbreeding–stress interactions across populations, we did see large differences in how populations responded to environmental stress. Furthermore, our results contrasted sharply with those of Fox et al. (2011), raising the question of whether stress responses may not only generalize poorly across populations, but across time as well. In light of these results, we suggest that for management purposes, generalizations about the effects of inbreeding–stress interactions across space (i.e., geography) and time (i.e., across many generations in a single population, especially when evolutionary rescue or purging is thought to be occurring) be approached with caution.

In summary, research over the past two decades has revealed substantial variation in magnitude and direction of inbreeding–stress interactions populations experience. Elucidating the various causes of such variation may help us not only to better predict a population's conservation risk, but to develop a deeper understanding of eco‐evolutionary dynamics as a whole. Until then, carefully considering the many nuances that can affect the magnitude and relevance of inbreeding–stress interactions may help us make sound judgments with regard to conservation.

## CONFLICT OF INTEREST

None declared.

## Data Availability

Data and computer code used to analyze these data will be archived in Dryad https://doi.org/10.5061/dryad.z8w9ghx8s.
